# Triple gallbladder with heterotopic gastric mucosa: a case report

**DOI:** 10.1186/s12887-022-03122-7

**Published:** 2022-01-20

**Authors:** Leah Ott, John O’Neill, Danielle Cameron, Michael J. Callahan, Amit Grover, Victor L. Fox, Heung Bae Kim, Craig Lillehei, Alex G. Cuenca

**Affiliations:** 1 Department of General Surgery, Boston Children’s Hospital, 300 Longwood Avenue, Fegan 3, Boston, MA 02115 USA; 2 Department of Pathology, Boston Children’s Hospital, Boston, MA 02115 USA; 3 Department of Radiology, Boston Children’s Hospital, Boston, MA 02115 USA; 4 Division of Gastroenterology, Hepatology, & Nutrition, Boston Children’s Hospital, Boston, MA 02115 USA

**Keywords:** Triple gallbladder, Multiple gallbladders, Heterotopic gastric mucosa

## Abstract

**Background:**

Triple gallbladder is a rare congenital anomaly of the biliary tract that can be associated with heterotopic tissue. Gallbladder triplication results from the failure of rudimentary bile ducts to regress during embryological development, and can be difficult to distinguish from Todani type II choledochal cysts and biliary duplication cysts.

**Case presentation:**

A 2-year-old patient presented to our institution with intermittent abdominal pain for 1 year. She had elevated transaminases with imaging concerning for a choledochal cyst. After assessment with magnetic resonance cholangiopancreatography and endoscopic retrograde cholangiopancreatography, she was diagnosed with a gallbladder multiplication and a common bile duct stricture. She underwent laparoscopic cholecystectomy, which confirmed the diagnosis of triple gallbladder. One of the three gallbladders demonstrated heterotopic gastric mucosa on final pathology, including at the cystic duct margin. Follow up testing with a technetium 99 m scan demonstrated a subtle focus of increased activity in the right upper abdomen at the expected location of the common bile duct, concerning for the presence of residual gastric mucosa. The patient remains well without abdominal pain.

**Conclusions:**

We describe the first case of heterotopic gastric mucosa in a triple gallbladder in a young patient presenting with chronic abdominal pain. We also demonstrate the safety and feasibility of laparoscopic cholecystectomy in young children with triple gallbladder. Finally, we propose an interdisciplinary approach to the management of common bile duct strictures in the setting of ectopic acid secretion, involving a combination of medical management, endoscopic intervention, and possible salvage laparoscopic Roux-en-Y hepaticojejunostomy.

## Background

Triple gallbladder is a rare congenital anomaly of the biliary system, resulting from the failure of rudimentary bile ducts to regress during the fifth and sixth weeks of gestation [1]. There have been several reported cases of triple gallbladder, which are typically managed with surgical excision given the high rate of concurrent pathology or malignancy [2]. Triple gallbladder can be difficult to distinguish from type II choledochal cysts, which may occur due to a similar process of over-proliferation of the bile duct epithelium during embryological development, but may result from several other mechanisms as well [3–7].

A second rare aberration of the biliary tract is the presence of heterotopic gastric mucosa, which may develop at multiple sites, such as the gallbladder, cystic duct, common hepatic duct, common bile duct (CBD), or ampulla of Vater [8]. Though heterotopic gastric mucosa in the biliary system and gallbladder multiplications are typically found separately, there is one report of heterotopic gastric mucosa lining a duplicate gallbladder [9]. We report the first case of heterotopic gastric mucosa in a triple gallbladder in a 2-year-old patient presenting with 1 year of abdominal pain. We also demonstrate the safety and efficacy of the laparoscopic cholecystectomy for the treatment of gallbladder multiplication in young children, and discuss the challenges of managing biliary strictures in the presence of heterotopic gastric mucosa.

## Case presentation

A 2-year-old female with a history of gastroesophageal reflux disease presented to an outside hospital with 1 year of intermittent abdominal pain, which had acutely worsened over 1 week. Her episodes of pain were associated with large volume, non-bilious emesis. Pain occurred almost daily, but had decreased in frequency after starting omeprazole. There was no family history of biliary disease. She had been evaluated at an outside hospital gastroenterology clinic 6 days prior, and labs demonstrated elevated alanine aminotransferase (ALT) 359 U/L, aspartate aminotransferase (AST) 146 U/L, alkaline phosphatase (ALP) 549 U/L, and gamma-glutamyl transpeptidase (GGT) 515 U/L, with a normal total bilirubin 0.2 mg/dL and lipase 25 U/L. Complete blood count and chemistry were unremarkable. Given her elevated transaminases, a right upper quadrant sonogram was obtained, which demonstrated biliary dilation and a saccular structure concerning for choledochal cyst. Upon presentation to the outside hospital, the patient appeared well and was afebrile and hemodynamically normal. Her abdomen was soft and non-tender.

**Fig. 1:**
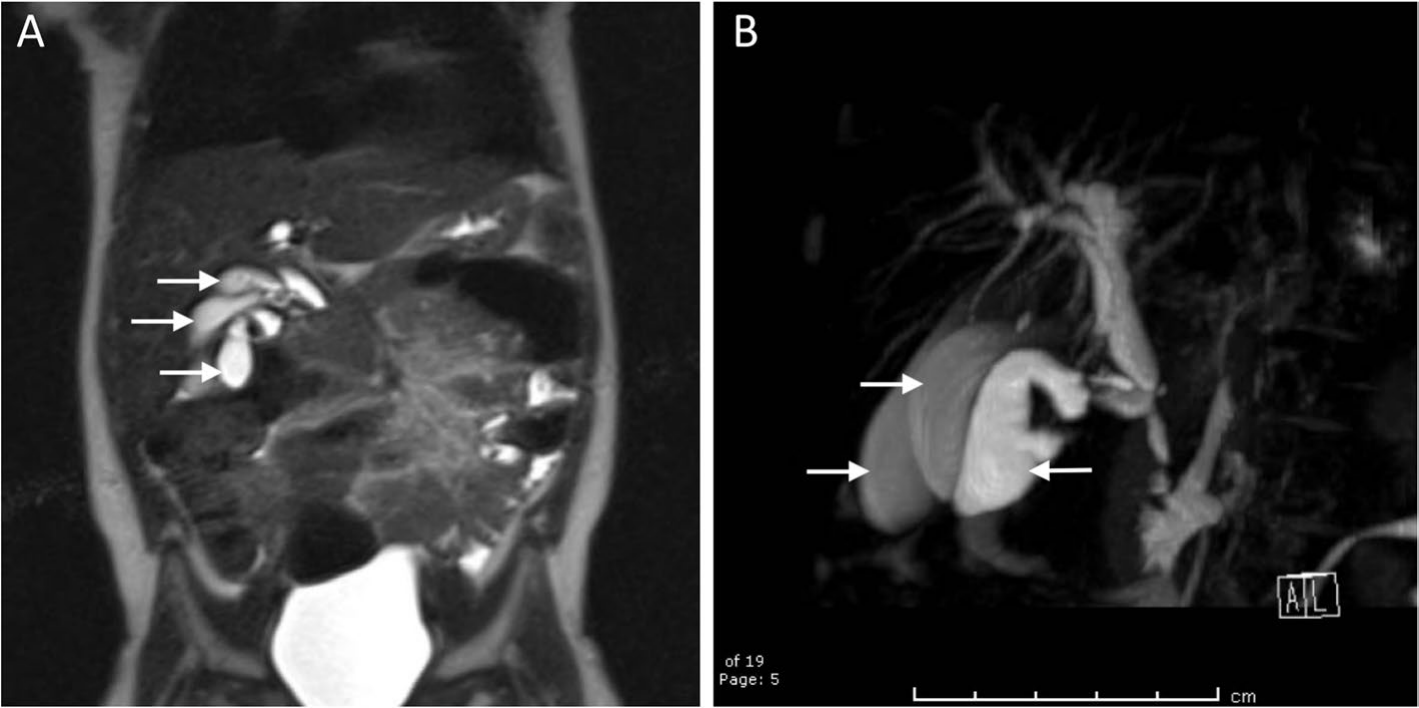
**A** Coronal view of preoperative magnetic resonance cholangiopancreatography demonstrating the gallbladder and two additional cystic structures in the gallbladder fossa (white arrows) communicating with biliary tract. **B** Oblique view of biliary tree reconstruction

Admission labs at the outside hospital were notable for elevated ALT 749 U/L, AST 973 U/L, and ALP 721 U/L, with a normal total bilirubin 0.5 mg/dL. Complete blood count and chemistry were within normal limits. The patient was admitted to the pediatrics floor and started on intravenous piperacillin-tazobactam, given the concern for ascending cholangitis. A repeat right upper quadrant sonogram was obtained, demonstrating intrahepatic and extrahepatic biliary dilation (with the common hepatic duct measuring 5.4 mm), as well as a tubular cystic structure anterior to the gallbladder that communicated with the common bile duct. Given the concern for type IV choledochal cyst, magnetic resonance cholangiopancreatography (MRCP) was obtained. This showed mild dilation of the central intrahepatic biliary ducts and two tubular cystic structures superior to the gallbladder in the gallbladder fossa, which were communicating with the common bile duct, as demonstrated in Fig. 1. These cystic structures measured 5 cm in length by 1.3 cm in diameter and 6 cm in length by 1.6 cm in diameter. The patient was evaluated by the Pediatric Surgery and Gastroenterology teams during her admission and given her clinical improvement and down-trending liver function tests, she was discharged home on hospital day three with a course of oral ciprofloxacin and a referral to our institution’s Pediatric Surgery clinic.

**Fig. 2:**
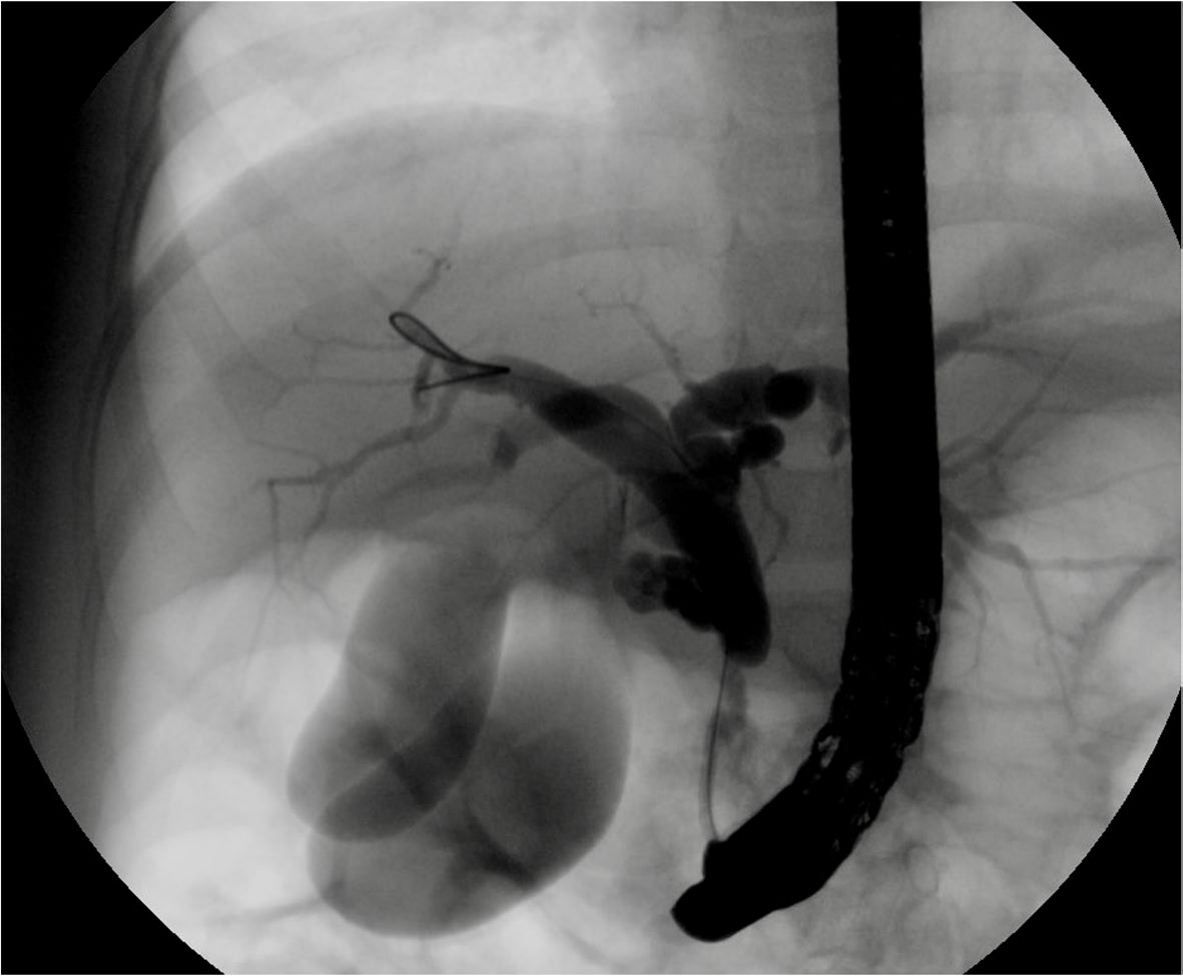
ERCP fluoroscopy image demonstrating opacification of two separate lumens, consistent with gallbladder duplication

Upon presentation to Pediatric Surgery clinic, the patient was doing well with only one episode of abdominal pain since discharge from the hospital 3 days prior. Given the concern for type IV choledochal cyst versus duplicated gallbladder, the case was discussed with the Gastroenterology team and the decision was made to proceed to endoscopic retrograde cholangiopancreatography (ERCP). This was notable for diffuse dilation of the common hepatic duct up to and involving the hepatic duct bifurcation, left main hepatic duct, and right hepatic duct on fluoroscopy. The junction between the pancreatic duct and CBD appeared normal. There was also filling of two separate cystic structures, consistent with two separate gallbladders, as seen in Fig. 2. A cholangiogram was performed, demonstrating narrowing of the distal CBD transitioning abruptly to the dilated proximal CBD, suggestive of congenital web versus stenosis somewhere between the ampulla and the cystic duct. This stenosis was dilated to 4 mm with good effect. After discussion with the Gastroenterology team, the decision was made to decompress the biliary system with plans for interval laparoscopic cholecystectomy. A 7 French, 5 cm biliary stent was placed in the CBD, with good flow of bile at the end of the procedure. She was scheduled for surgery 11 days later.

**Fig. 3:**
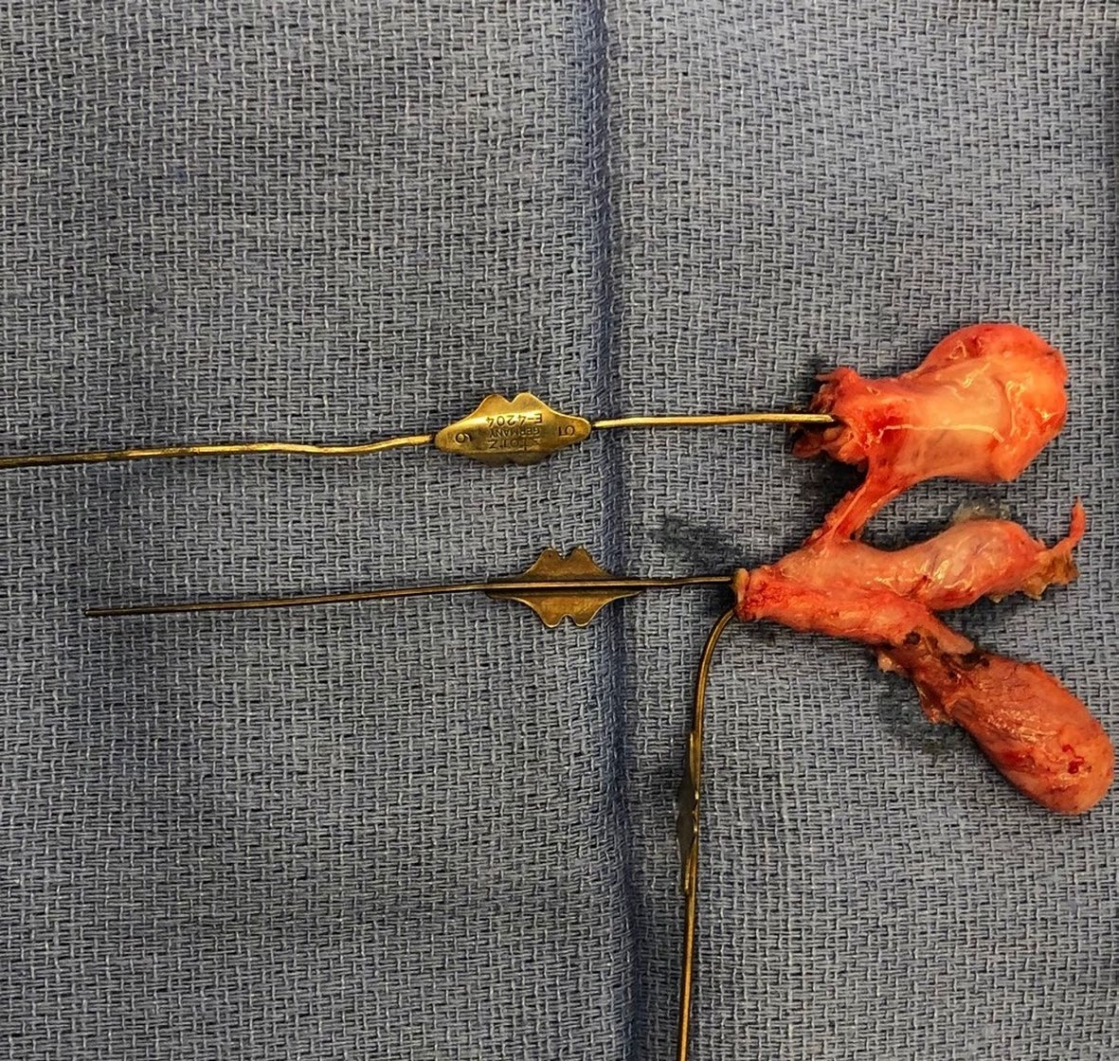
Triple gallbladder surgical specimen, with lacrimal probes within each cystic duct

During the laparoscopic cholecystectomy, what appeared to be two separate cystic ducts were identified draining into two separate cystic structures, presumed to be unique gallbladders. Due to the aberrant anatomy, the cystic structures were dissected free from the cystic plate with a dome down approach. No additional ducts or vascular structures were identified. Both cystic ducts and a single cystic artery were clipped and cut. Both ducts were reinforced with an Endoloop, given the need for future endoscopies for her congenital stricture. Once the specimen was opened on the back table, further dissection revealed what appeared to be three lumens of three cystic structures, as seen in Fig. 3. There appeared to be three separate cystic ducts, and lacrimal probes were passed through these ducts demonstrating three unique lumens, consistent with type 1 triple gallbladder. The surgical clips sufficiently crossed each of the three cystic ducts. The patient tolerated the procedure well and was discharged home on postoperative day one. Outpatient labs on postoperative day seven demonstrated normalization of her transaminases to ALT 14 U/L, AST 27 U/L, and ALP 218 U/L, with normal total bilirubin 0.3 mg/dL and direct bilirubin 0.01 mg/dL. Repeat right upper quadrant sonograms demonstrated resolution of the intrahepatic and extrahepatic biliary ductal dilation.

**Fig. 4:**
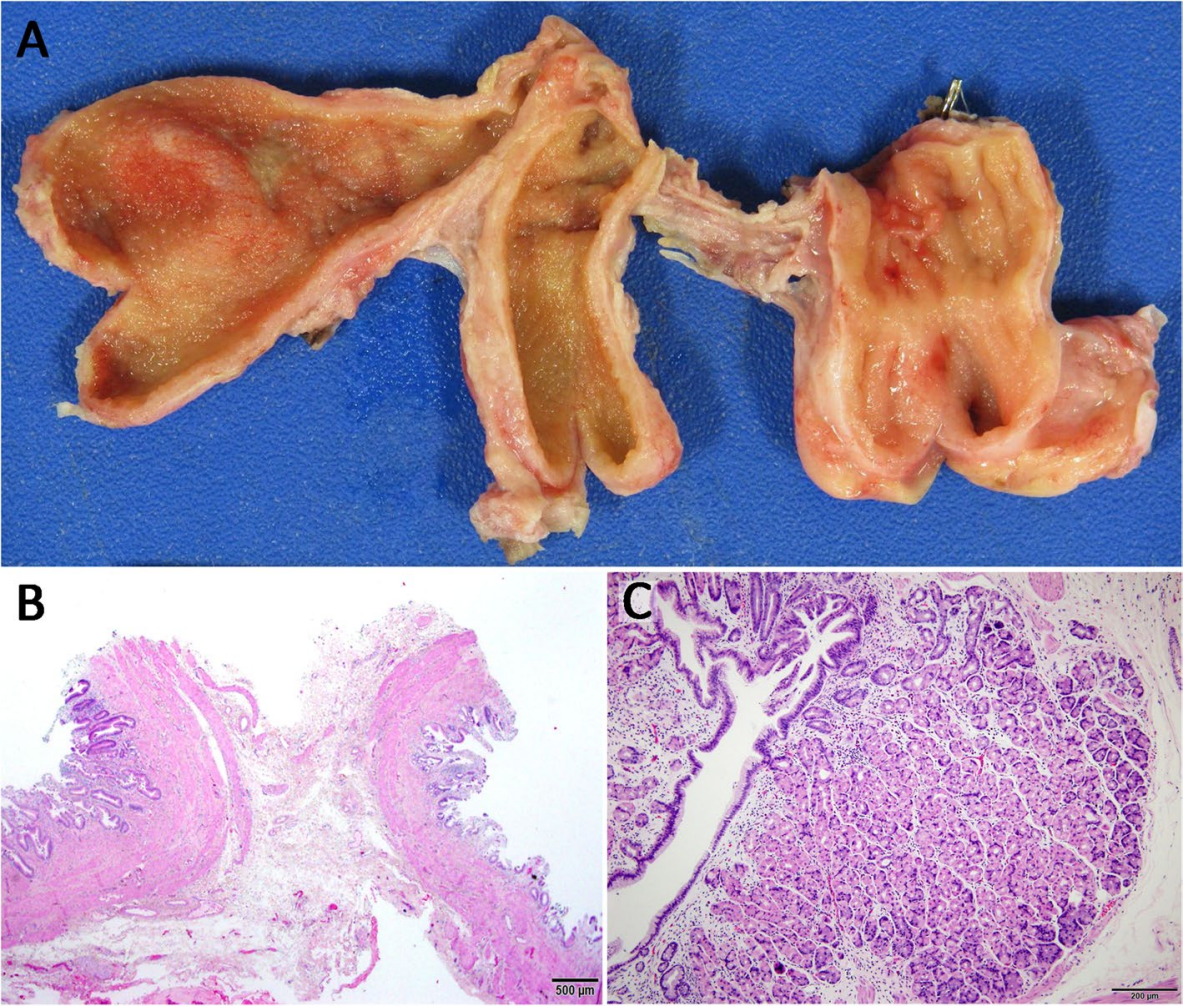
**A** Formalin fixed cholecystectomy specimen with lumens exposed. Conjoined gallbladders 1 and 2 (left and center) showed typical tan velvety biliary type mucosa, whereas attached mass lesion (right) notable for luminal gastric features; pale coarse rugal folds. **B** Hematoxylin and eosin (H&E) stained microscopic section (20X magnification) demonstrating back-to-back walls of conjoined gallbladders 1 and 2 with typical biliary type mucosa. Mural thickening is present indicating chronic cholecystitis. **C** Vast majority of separately attached lesion lined by oxyntic (body/fundal) type gastric mucosa (H&E, 100X magnification). Microscopic images obtained using Olympus® BX43 microscope with Olympus® DP27 camera and Olympus® cellSens Entry 2.3 (Build 18,987) image processing software

Pathologic examination of the cholecystectomy specimen (Fig. 4**A**) noted a triluminal structure composed of two conjoined specimens grossly lined by biliary mucosa (left and center). A third specimen (right), attached to the remaining specimen by a delicate fibrous band, harboured features of gastric type mucosa. Histopathological examination confirmed the gross impression wherein the conjoined specimens showed typical biliary mucosa (Fig. 4**B**) and chronic cholecystitis. Additionally, the third specimen was almost entirely lined by oxyntic type gastric mucosa (Fig. 4**C**) typically found in the gastric body and fundus. The patient’s pathology was determined to be most consistent with triple gallbladder, although duplicated gallbladder with an attached biliary duplication cyst could not be definitively excluded.

Heterotopic gastric mucosa was also present at the cystic duct margin of the third specimen, and the patient was continued on omeprazole postoperatively. Five months following cholecystectomy, her biliary stent was removed. Approximately 10 months after cholecystectomy, she underwent a technetium 99 m scan, which demonstrated a subtle focus of increased activity in the right upper abdomen at the expected location of the common bile duct, concerning for the presence of residual gastric mucosa. Following discussion between the Surgery and Gastroenterology teams, the decision was made to manage the patient expectantly, with acid suppression with omeprazole and serial labs and sonograms to assess for recurrence of her congenital CBD stricture. Should this stricture recur, it will be managed endoscopically, with possible future laparoscopic Roux-en-Y hepaticojejunostomy for salvage should endoscopic management fail in the setting of ongoing ectopic acid secretion. The patient currently remains well and without abdominal pain.

## Discussion and conclusions

Triple gallbladder (vesica fellae triplex) was first described by Huber in a human cadaver in 1752 [10]. It is a rare congenital anomaly of the biliary tract, with 17 reported cases. Triple gallbladder occurs due to failure of rudimentary bile ducts to regress during the fifth and sixth weeks of gestation, creating outpouchings in the biliary system that develop into accessory gallbladders [1]. Three types of triple gallbladder have been described, based on variable anatomy of the cystic duct. Type 1 occurs when each gallbladder has its own cystic duct directly draining into the common bile duct, as seen in this case. Type 2 occurs when two gallbladders share a common cystic duct, while the third drains via a separate cystic duct. Finally, in type 3, all three gallbladders drain via a single cystic duct. The type of triple gallbladder appears to depend on the location of these outpouchings along the biliary system during embryological development. If these buds occur along the common bile duct or common hepatic duct, then separate cystic ducts will develop; if buds develop in the cystic duct, a shared common cystic duct will form [11]. Gallbladder multiplications can be difficult to distinguish from Todani type II choledochal cysts, which are described as congenital diverticular malformations of the common bile duct [12]. Todani type II choledochal cysts may result from a similar process of excessive epithelial proliferation during the solid bile duct stage, but several other mechanisms have also been proposed, including irregular bile duct recanalization, reflux of pancreatic secretions, weakness or structural defects of the bile duct wall, or defects in the inferior choledochal sphincter leading to high intraductal pressures [3–7, 13, 14]. Rarely, Todani type I choledochal cysts and duplicate gallbladder have been discovered concurrently [15–17].

Heterotopic gastric mucosa in the biliary system is another congenital anomaly, and has been identified at multiple sites, such as the ampulla of Vater, common bile duct, common hepatic duct, cystic duct, and gallbladder [8]. Although various heterotopic tissues, such as adrenal, thyroid, pancreas, liver, and small intestine, have been isolated from the biliary tract, gastric mucosa remains the most common [18–20]. Heterotopic gastric mucosa is most often fundic type, as in this case, featuring chief and parietal cells [21]. Most cases of heterotopic gastric mucosa in the biliary tree are asymptomatic, but may present with symptoms secondary to gallbladder inflammation, obstruction, or perforation [18, 22–25]. Rarely, obstructive jaundice or hemobilia have been observed [25, 26]. Mucosal ulceration is uncommon, likely due to the presence of alkaline bile [18].

Herein, we present the youngest case of triple gallbladder and the first instance of heterotopic gastric mucosa in a triple gallbladder. Bailie et al. described a case of a 7-year-old child who initially presented with recurrent episodes of pancreatitis and was found to have a duplicate gallbladder, which was removed via laparotomy with anatomy confirmed on intra-operative cholangiogram [9]. Pathological examination revealed one gallbladder with predominantly fundic type mucosa and some antral type mucosa, and evidence of chronic inflammation [9]. Her symptoms improved following cholecystectomy, although she continued to have bouts of mild pancreatitis for several months postoperatively [9]. There have also been cases of heterotopia in triple gallbladder, with Kelly describing a 12-year-old boy with heterotopic duodenal-type mucosa lining one of the three gallbladders [27]. The third structure lined by heterotopic gastric mucosa in this case could represent a biliary duplication cyst, but this seems to be a matter of semantics. There have also been reports of heterotopic gastric mucosa lining biliary duplication cysts [28, 29].

Preoperative diagnosis of triple gallbladder can be challenging, with imaging often failing to demonstrate all three gallbladders [30–32]. Chances of successful preoperative diagnosis increase when multiple imaging modalities, including ultrasonography, endoscopic ultrasonography, and MRCP, are utilized [33]. Multiple groups have previously shown the feasibility and safety of laparoscopic cholecystectomy for gallbladder multiplications in adolescent and adult patients, often with intra-operative cholangiogram to decrease the risk of damage to the biliary system [30, 31, 33–35]. Herein, we demonstrate that laparoscopic cholecystectomy is a safe and effective approach for the treatment of gallbladder multiplication in a 2-year-old child. Additionally, heterotopic gastric mucosa at the cystic duct margin and the suggestion of residual gastric tissue in the common bile duct on the technetium 99 m scan pose a challenge for future management. Based on interdisciplinary discussions, the patient’s common bile duct stricture will be managed endoscopically as she grows, temporizing it until salvage Roux-en-Y hepaticojejunostomy can feasibly be performed laparoscopically.

In summary, we described a novel case of a pediatric patient with heterotopic gastric mucosa in a triple gallbladder. Both heterotopic gastric mucosa in the biliary system and gallbladder multiplication are rare causes of abdominal pain in children, with the latter necessitating cholecystectomy to prevent or exclude malignancy in older patients. As previously shown with adolescents and adults, we demonstrated that laparoscopic cholecystectomy is a safe and effective surgical approach for the treatment of triple gallbladder in young children.

## Data Availability

All data generated or analysed during this study are included in this published article.

## Abbreviations

ALT: Alanine aminotransferase
AST: Aspartate aminotransferase
CBD: Common bile duct
Cm: Centimeters
ERCP: Endoscopic retrograde cholangiopancreatography
GGT: Gamma-glutamyl transpeptidase
Mg/dL: Milligrams/deciliter
Mm: Millimeters
MRCP: Magnetic resonance cholangiopancreatography
U/L: Units/liter
